# Large-scale network dynamics of beta-band oscillations underlie auditory perceptual decision-making

**DOI:** 10.1162/NETN_a_00009

**Published:** 2017-06-01

**Authors:** Mohsen Alavash, Christoph Daube, Malte Wöstmann, Alex Brandmeyer, Jonas Obleser

**Affiliations:** Department of Psychology, University of Lübeck, Germany; Max Planck Research Group “Auditory Cognition,” Max Planck Institute for Human Cognitive and Brain Sciences, Leipzig, Germany

**Keywords:** Network dynamics, Perceptual decision, Oscillation, MEG, Functional connectivity

## Abstract

Perceptual decisions vary in the speed at which we make them. Evidence suggests that translating sensory information into perceptual decisions relies on distributed interacting neural populations, with decision speed hinging on power modulations of the neural oscillations. Yet the dependence of perceptual decisions on the large-scale network organization of coupled neural oscillations has remained elusive. We measured magnetoencephalographic signals in human listeners who judged acoustic stimuli composed of carefully titrated clouds of tone sweeps. These stimuli were used in two task contexts, in which the participants judged the overall pitch or direction of the tone sweeps. We traced the large-scale network dynamics of the source-projected neural oscillations on a trial-by-trial basis using power-envelope correlations and graph-theoretical network discovery. In both tasks, faster decisions were predicted by higher segregation and lower integration of coupled beta-band (∼16–28 Hz) oscillations. We also uncovered the brain network states that promoted faster decisions in either lower-order auditory or higher-order control brain areas. Specifically, decision speed in judging the tone sweep direction critically relied on the nodal network configurations of anterior temporal, cingulate, and middle frontal cortices. Our findings suggest that global network communication during perceptual decision-making is implemented in the human brain by large-scale couplings between beta-band neural oscillations.

## Author Summary

The speed at which we make perceptual decisions varies. This translation of sensory information into perceptual decisions hinges on dynamic changes in neural oscillatory activity. However, the large-scale neural-network embodiment supporting perceptual decision-making is unclear. We addressed this question by experimenting two auditory perceptual decision-making situations. Using graph-theoretical network discovery, we traced the large-scale network dynamics of coupled neural oscillations to uncover the brain network states that support the speed of auditory perceptual decisions. We found that higher network segregation of coupled beta-band oscillations supports faster auditory perceptual decisions over trials. Moreover, when auditory perceptual decisions are relatively difficult, the decision speed benefits from higher segregation of frontal cortical areas, but lower segregation and greater integration of auditory cortical areas.

Contemporary neuroscience is departing from a focus solely on regional brain activations, toward emphasizing the network organization of brain function. This view recognizes the large-scale interactions between distributed cortical areas as the biological basis of behavior and cognition (Misic & Sporns, 2016; Sporns, 2014). A more mechanistic view holds frequency-specific neural oscillations to be relevant to both behavior and cognition (Buzsaki & Draguhn, 2004; Engel, Gerloff, Hilgetag, & Nolte, 2013). How the large-scale network organization of interacting neural oscillations (S. Palva & Palva, 2012; Siegel, Donner, & Engel, 2012)—in particular, their temporal network dynamics (Kopell, Gritton, Whittington, & Kramer, 2014; Shineet al., 2016)—relate to perception and cognition is poorly understood, however. Here we investigated the dependence of auditory perceptual decision-making in humans on spectrally, temporally, and topologically resolved large-scale brain networks.

Accumulating evidence suggests that frequency-specific neural oscillations are key to processing sensory information (de Pesters et al., 2016; Hanslmayr, Gross, Klimesch, & Shapiro, 2011; J. M. Palva, Monto, Kulashekhar, & Palva, 2010). For example, previous studies indicate that attentional modulation of cortical excitability in sensory regions is reflected in oscillatory alpha power (∼8–10 Hz) under visual (Jensen & Mazaheri, 2010; Lange, Oostenveld, &Fries, 2013; Lou, Li, Philiastides, & Sajda, 2014) or auditory tasks (Müller & Weisz, 2012; Strauß,Wöstmann, & Obleser, 2014; Weisz, Muller, Jatzev, & Bertrand, 2014; Wöstmann,Herrmann, Maess, & Obleser, 2016). Additionally, it has been shown that audiovisual perception relies on synchronized cortical networks within beta (∼20 Hz) and gamma (∼80 Hz) bands (Hipp, Engel, & Siegel, 2011). Recently, studies have begun to explore more specifically whether modulations in neural oscillations arise from lower-order sensory or higher-order control areas (Friese et al., 2016; Kayser, McNair, & Kayser, 2016; Park, Ince, Schyns, Thut, &Gross, 2015; Sadaghiani & Klenschmidt, 2016). Here, on the basis of localization of neurophysiological sources (Hillebrand, Singh, Holliday, Furlong, & Barnes, 2005), we explored the large-scale network organization of interacting neural oscillations during auditory processing.

Specifically, we asked how the network topology of coupled neural oscillations (Bassett et al., 2009) relates to the listeners’ perceptual decisions. In a previous magneto encephalography (MEG) study, Nicol and colleagues (2012) measured the synchronization of brain gamma-band (33–64 Hz) responses in an auditory mismatch-negativity paradigm. They found that deviant stimuli were associated with increases in local network clustering in left temporal sensors within the immediate response period. Building upon prestimulus hemodynamic responses, Sadaghiani, Poline, Kleinschmidt, and D’Esposito (2015) recently suggested higher modularity of brain networks as a proxy for perceiving near-threshold auditory tones. Moreover, it has been shown that higher global integration of brain networks measured from prestimulus high-alpha band MEG responses precedes the detection of near-threshold stimuli (Leske et al., 2015; Weisz, Wuhle et al., 2014). In sum, brain network correlates of auditory perception have been observed on different topological scales.

Naturally, the cortical networks involved in processing sensory information require context-sensitive configurations, as well as moment-to-moment reconfigurations to fulfill dynamic task adjustments (Bassett, Meyer-Lindenberg, Achard, Duke, & Bullmore, 2006). This leads the neural coactivations, which shape the brain’s functional connectivity, to diverge from their underlying structural connectivity (Marrelec, Messe, Giron, & Rudrauf, 2016; Misic et al., 2016; Park & Friston, 2013). As such, the estimation of functional connectivity, when collapsed over time, overemphasizes structural connectivity (Honey, Kotter, Breakspear, & Sporns, 2007; Shen, Hutchison, Bezgin, Everling, & McIntosh, 2015) and disregards the temporal dynamics of large-scale brain network topology (Kringelbach, McIntosh, Ritter, Jirsa, & Deco, 2015; Zalesky, Fornito, Cocchi, Gollo, & Breakspear, 2014). However, these dynamics have been found relevant to behavior during challenging motor or cognitive tasks (Alavash, Thiel, & Giessing, 2016; Bassett et al., 2013; Braun et al., 2015; Chai, Mattar, Blank, Fedorenko, & Bassett, 2016).

Therefore, to find the neural network substrate of auditory perceptual decision-making, we adopted the framework of dynamic brain networks (Calhoun, Miller, Pearlson, & Adali, 2014; Deco, Tononi, Boly, & Kringelbach, 2015) and merged this with neural oscillations to uncover frequency-specific brain network states. Our method is based on both a previously established technique to estimate large-scale neural interactions in source space (Hipp, Hawellek, Corbetta, Siegel, & Engel, 2012) and graph-theoretical network analysis (Bullmore & Sporns, 2009).

**Figure 1.**
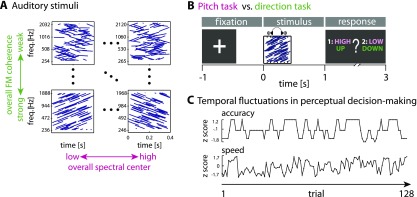
Experimental stimuli and tasks. (A) Auditory stimuli used to design the tasks. Each cell represents an acoustic texture, which can be viewed as a pattern of sound sweeps whose frequency increases or decreases over time. Each texture stimulus had a duration of 400 ms and consisted of 72 frequency-modulated (FM) sweeps of 100-ms duration. The stimuli were titrated along two dimensions: overall coherence and spectral center. For a given stimulus, a variable proportion (25–100%) of the sweeps were assigned the same frequency slope (coherence)—that is, their frequency went up or down at the same rate over time. The rest of the sweeps had a randomly assigned slope. In addition, each stimulus had one of eight spectral centers relative to a mean center frequency. (B) Two auditory perceptual decision-making tasks, namely *pitch* and *direction* discrimination, were designed using the identical acoustic stimuli. During the pitch task, the subjects judged the overall pitch of the stimuli (low or high). In the direction task, they were asked to report the overall direction (up or down) in which the frequencies of the stimuli were changing (increasing or decreasing) over time. Subjects had 3 s at maximum to press one of two response buttons to report their perceptual decision. In each task, the decision labels for the left-hand and right-hand buttons (indicated by “1”/“2”) were randomized across trials and shown after stimulus pre sentation within the response window. There were eight blocks per task, and the order of the tasks alternated from one block to another. (C) Exemplary trial-by-trial auditory perceptual decision accuracy (moving average of four trials applied to correct/incorrect responses) and decision speed ([response time]^−1^). Before the actual tasks, adaptive perceptual tracking was used to tailor the two tasks per participant, so that their overall accuracies converged at ∼70%. This led individuals’ decision accuracies and speeds to fluctuate over trials. Note that for the purpose of the regression analysis (see Materials and Methods), the trial-wise estimates of accuracy and speed were first rank-transformed and then normalized (i.e., *z*-scored). Exemplary data are shown for a representative participant in the pitch task, second block.

**Figure 2.**
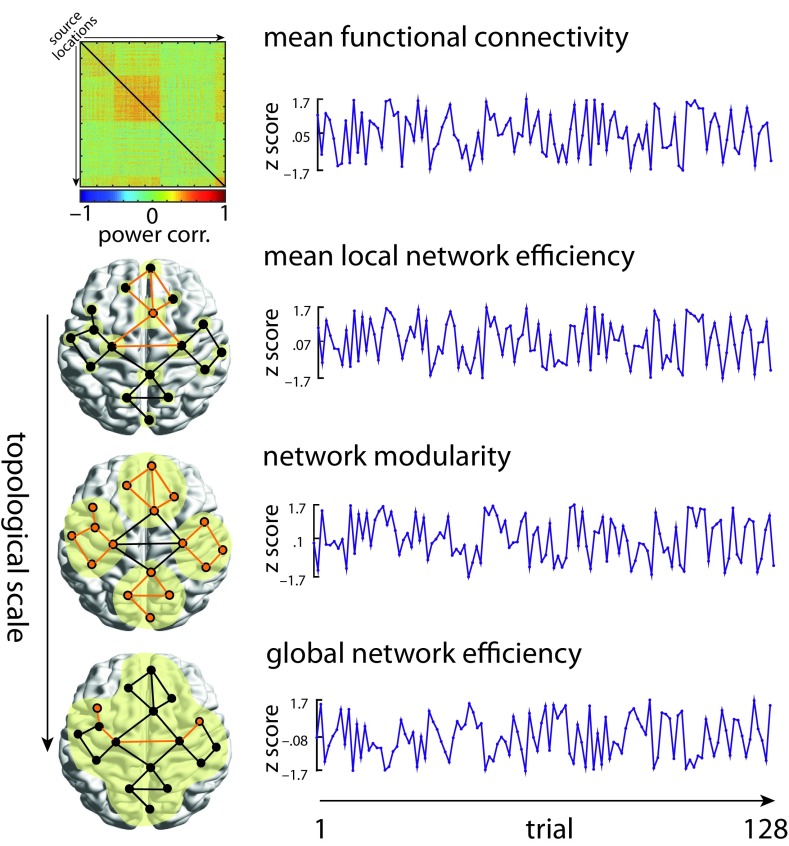
Trial-by-trial dynamics of brain functional connectivity and network topology. To investigate the relation between the frequency-specific brain network states and decision-making performance, all-to-all power-envelope correlations (source connectivity matrix; top left) and whole-brain graph-theoretical network metrics were estimated at 10% of the network density (see Materials and Methods). This analysis was done at each frequency within 1–32 Hz, and per trial in the course of each pitch and direction task. The temporal graph-theoretical metrics captured brain network states on the local (local efficiency), intermediate (modularity), and global (global efficiency) scales of network topology. The yellow-shaded ovals in brain graphs illustrate the topological scale at which each network metric is measured. *Global efficiency* (bottom graph) is inversely related to the sum of shortest path lengths (e.g., orange path) between every pair of nodes. *Meanlocal efficiency* (top graph) is equivalent to global efficiency computed on the direct neighbors of each node (e.g., the orange node), which is then averaged over all nodes. *Modularity* (middle graph) describes the segregation of partner nodes into relatively dense groups (here, the orange nodes forming four modules), which are sparsely interconnected. For the purpose of the regression analysis (see Materials and Methods), the trial-wise estimates of network metrics were first rank-transformed and then normalized (i.e., *z*-scored). Exemplary data are shown for a representative subject, as in Figure 1C.

We applied this approach to MEG signals measured from human listeners who made perceptual decisions on brief acoustic textures under two distinct task sets. The acoustic textures consisted of densely layered tone sweeps that varied in their overall pitch (high or low) as well as in the proportion of coherent tones in terms of sweep direction (up or down; Figure 1A). Using the identical set of stimuli, two auditory paradigms with distinct decision contexts were designed to deliver challenging perceptual decision-making tasks (Figure 1B). As such, the individuals’ perceptual decision accuracy and speed fluctuated on a trial-by-trial basis (Figure 1C). This allowed us to investigate the relation between frequency-specific brain network states and trial-by-trial decision-making performance (Figure 2). Since, under each of the two perceptual decision-making tasks, subjects focused on a different acoustic feature of an identical set of auditory stimuli, two dynamic network profiles were expected. First, we anticipated that the brain network states responsible for the cortico-cortical communication (mainly frontotemporal) involved in common for both of the tasks would predict decision-making performance. Second, we expected that any task-specific brain network states emerging from auditory association or higher-order decision areas would differentially predict performance in either of the tasks.

## RESULTS

### Auditory Perceptual Decision-Making Performance

The participants judged the overall pitch or sweep direction of the acoustic texture stimuli and showed accuracies around 70%, as we had intended from use of the adaptive-tracking procedure (average accuracies ± *SEM*s: pitch task = 76% ± 1.1, direction task = 70% ± 2.1; mean decision speed [s^−1^] ± *SEM*s: pitch task = 1.8 ± 0.1, direction task = 1.7 ± 0.1). The bootstrap Kolmogorov–Smirnov test revealed that the distributions of the behavioral measures did not significantly deviate from a normal distribution (accuracy: pitch task *p* = 0.8, direction task *p* = 0.1; decision speed: pitch task *p* = 0.8, direction task *p* = 0.5). Despite our experimental efforts to equate the difficulties of both tasks, analysis of variance (ANOVA) revealed main effects of task for both accuracy [*F*(1, 19) = 6, *p* = 0.024, $\eta_{G}^{2}$ = 0.05] and decision speed [*F*(1, 19) = 9.9, *p* = 0.005, $\eta_{G}^{2}$ = 0.01]. Participants showed significantly lower accuracy in the direction task than in the pitch task (exact permutation test for a paired test; *p* = 0.02). In addition, the decision speed was significantly lower in the direction task than in the pitch task (*p* = 0.004). The experimental manipulation of the pitch of the acoustic textures yielded significant effects of stimulus spectral center on both accuracy [*F*(3, 57) = 64, *p* = 2*e*−16, $\eta_{G}^{2}$ = 0.18] and decision speed [*F*(3, 57) = 30, *p* = 9*e*−12, $\eta_{G}^{2}$ = 0.02]. However, stimulus coherence had a significant effect only on accuracy [*F*(3, 57) = 45.3, *p* = 4*e*−15, $\eta_{G}^{2}$ = 0.12]. Across participants, we found a significant positive correlation between decision speed in the pitch task and decision speed in the direction task (Spearman’s *ρ* = 0.9, *p* = 5*e*−6). However, the correlation between the accuracies in the two tasks was not significant (*ρ* = 0.2, *p* = 0.47).

The correlation between the trial-by-trial estimates of decision accuracy or speed and the trial-by-trial acoustic features of the stimuli was tested using a two-level regression analysis (see Materials and Methods). We found a significant positive correlation between decision speed and stimulus coherence in the direction task (average regression weights ± *SEM*s: 0.045 ± 0.012; one-sample exact permutation test: *p* = 9*e*−4). The trial-by-trial estimates of decision accuracy in the direction task also showed a significant positive correlation with the coherence of the stimuli (average regression weights ± *SEM*s: 0.087 ± 0.01, *p* = 2*e*−6). A significant negative correlation emerged between decision accuracy in the pitch task and stimulus coherence (average regression weight ± *SEM*: −0.022 ± 0.01, *p* = 0.03).

### Neural Oscillatory Power During Auditory Perceptual Decision-Making

**Figure 3.**
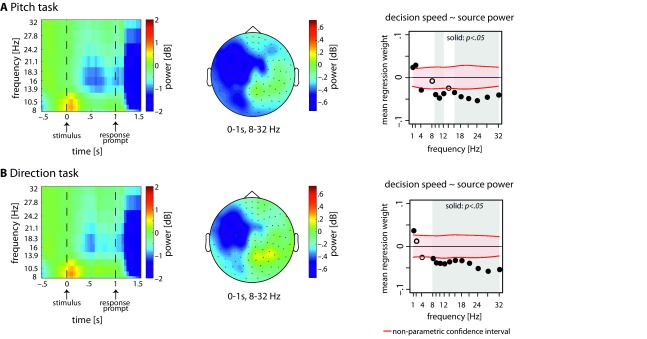
Dynamics of neural oscillatory power under each auditory task and their relation to the speed of perceptual decisions. (Left) Spectrotemporal representations of the epoched signals during (A) the pitch task and (B) the direction task were estimated and averaged over trials, 102 combined gradiometer sensors, and all subjects (*N* = 20). Whereas an increase in oscillatory alpha (8–13 Hz) power was observed time-locked to the auditory stimulation relative to the baseline interval (–0.5 to 0 s), there was a decrease in oscillatory power within the low- and mid-beta bands (14–24 Hz) during the time window in which participants listened to the stimuli (0 to 1 s). (Middle) The topographical maps show the broadband baseline-corrected oscillatory power (8–32 Hz) from stimulus onset to the onset of the response prompt (0–1 s). Note that within this time period, the subjects were not yet aware of the mapping between the decision labels (pitch task: high/low; direction task: up/down) and the left-hand/right-hand response buttons, since the mapping was randomized across trials. (Right) Results from the regression analysis. The relation between the ongoing power of neural oscillations and decision speed was investigated by means of linear regressions in which the trial-by-trial decision speed was predicted by the oscillatory power of source signals. In the course of both auditory tasks, faster perceptual decisions were negatively correlated with the ongoing oscillatory power of source signals within the alpha and beta bands. The black circles show normalized regression weights averaged over subjects at each frequency. The horizontal shaded regions show 95% confidence intervals of the null mean regression weights generated by circularly shifting the behavioral responses across trials (corrected for multiple comparisons across frequency bins using the false-coverage statement rate [FCR]; *p* < 0.05).

We investigated power perturbations in the MEG oscillatory signal while subjects listened to the acoustic textures and judged either their overall pitch or sweep direction. As is illustrated in Figure 3, MEG oscillatory alpha (∼8–13 Hz) power was increased relative to the baseline interval (–0.5 to 0 s) just after stimulus presentation. In addition, during and after stimulus presentation but before the response prompt (0 to 1 s), we observed left-lateralized decrease in MEG oscillatory power in the low- and mid-beta band (∼14–24 Hz) relative to the baseline interval. The above perturbations in alpha and beta bands were similarly observed in both the pitch and direction tasks (Figures 3A and 3B, first two panels) and are well in line with previous studies on the neural substrates of perceptual decision-making (Donner, Siegel, Fries, & Engel, 2009; Haegens et al., 2011; Kelly & O’Connell, 2015; O’Connell, Dockree, & Kelly, 2012). Finally, as expected, there was strong, motor-related suppression in the MEG oscillatory power relative to baseline within the time interval when the subjects manually reported their perceptual decision following the response prompt (Pfurtscheller & Lopes da Silva, 1999). This perturbation was widely distributed within the alpha and beta bands (8–32 Hz; Figures 3A and 3B, first panels).

Since the aim of this study was to find the relation between frequency-specific brain network states and auditory perceptual decision-making on a trial-by-trial basis, we next focused on trial-by-trial fluctuations in the power of the source-projected signals. Toward this end, we implemented a general linear model (GLM) per participant, whereby the time series estimates of trial-by-trial decision accuracy or speed (Figure 1C) were predicted by the baseline-corrected power of the whole-brain source-projected signals. We found significant negative correlations between decision speed during either the pitch or direction task and ongoing neural oscillatory power within the alpha and beta bands (8–32 Hz; Figures 3A and 3B, third panels). These results indicate that, during both the pitch and direction tasks, a stronger decrease in neural oscillatory power relative to the baseline interval correlated with faster perceptual decisions. However, we found no significant correlation between decision accuracy and the ongoing oscillatory power of the source-projected signals.

### Whole-Brain Network Dynamics of Beta-Band Oscillations Predict Decision Speed

The aim of this study was to find the frequency-specific brain network states underlying individuals’ perceptual decision-making in the course of judging auditory stimuli. The auditory stimuli were identical but were presented in two distinct task sets—that is, either judging the overall pitch or the overall direction of frequency-modulated tone sweeps. To predict trial-by-trial decision-making performance from the ongoing brain network states, we implemented a linear regression model in which the time series estimates of trial-by-trial decision accuracy or speed (Figure 1C) were predicted by temporal graph-theoretical network metrics (Figure 2).

**Figure 4.**
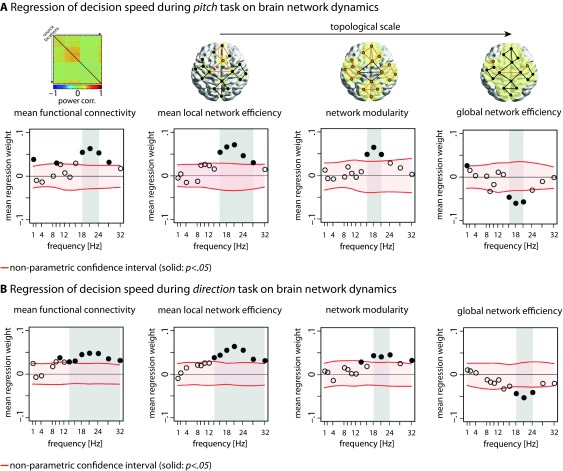
Whole-brain network dynamics of beta-band oscillations predicted decision speed. The relation between the ongoing dynamics of large-scale brain networks and perceptual decisions on auditory stimuli was investigated by means of linear regressions in which trial-by-trial decision speed was predicted by temporal graph-theoretical network metrics. This analysis was done separately for each (A) pitch and (B) direction task, at frequencies ranging from 1 to 32 Hz. In the course of both auditory tasks, faster perceptual decisions positively correlated with the ongoing local efficiency (second column) and modular segregation (third column) of brain networks built upon beta-band oscillations. However, higher global integration showed the opposite effect (fourth column). Black circles indicate the mean regression weights averaged over subjects at each frequency. Horizontal shaded regions show 95% confidence intervals of the null mean regression weights generated by circularly shifting the behavioral responses across trials (corrected for multiple comparisons across frequency bins using the false-coverage statement rate; *p* < 0.05). The graphs at top provide schematic illustrations of graph-theoretical metrics capturing brain network states at the local (local efficiency), intermediate (modularity), and global (global efficiency) scales of topology. The yellow shaded ovals illustrate the topological scale at which each network metric is measured.

On the whole-brain level, for both the pitch and direction tasks, we found significant cor relations between decision speed, on the one hand, and the functional connectivity and topology of dynamic brain networks, on the other hand (Figure 4). These correlations peaked within the beta-band range (Figure 4, solid points). The significant correlations indicate that, for both the pitch and direction tasks, higher local efficiency but lower global efficiency of large-scale brain networks supported faster perceptual decisions (Figure 4, second and last columns, respectively). In addition, higher segregation of brain network modules predicted faster perceptual decisions in both tasks (Figure 4, third column).

More specifically, we found positive correlations between the mean functional connectivity of dynamic brain networks and decision speed in both the pitch and direction task within the frequency range of 16 to 28 Hz (Figure 4, first column). In both tasks, faster perceptual decisions about the acoustic textures were accompanied by increases in the mean functional connectivity of dynamic brain networks over trials. This effect was not limited to functional connectivity, but was also reflected in the topology of dynamic brain networks. On the local scale of network topology, higher mean local efficiency of dynamic brain networks within the frequency range of 16 to 28 Hz predicted faster decisions in both tasks over trials (Figure 4, second column). Moreover, on the intermediate level of network topology, higher modularity of dynamic brain networks at the same frequencies also predicted faster decisions in both tasks (Figure 4, third column). Finally, on the global scale of network topology, faster decisions were predicted by decreases in global network efficiency at frequencies ranging from 18 to 21 Hz (Figure 4, fourth column1). Note that we did not observe any significant correlation between the trial-by-trial estimates of decision accuracy and brain network metrics in either of the tasks.

The analysis of neurobehavioral correlations was based on estimating all-to-all source connectivity per trial, which covered the time points from −0.5 to 1.5 s in steps of 0.05 s. To further investigate possible predictions from pre- or poststimulus dynamic network states, we applied the same analyses to the data measured during the prestimulus interval (−0.85 to 0 s) or the poststimulus interval (0 to +1 s) separately. In addition, to examine the extent to which our results might merely reflect neural processes involved in giving manual responses after the response prompt (see Figure 3), we also analyzed the mid-beta-band (16–28 Hz) power correlations using only the data measured during the response window ( +1 to +1.5 s). None of these analyses revealed consistent significant correlations with the speed of auditory perceptual decisions (Alavash, Daube, Wöstmann, Brandmeyer, & Obleser, 2017, Figure S1).

To test the task specificity of the correlations between a given network diagnostic and trial-by-trial decision accuracy or speed, we also computed mean differences in the regression weights. We found no significant difference between the two tasks in predicting decision accuracy or speed from the ongoing dynamics of network topology on the whole-brain level. In addition, to investigate the possible lead/lag relationship between brain network states and trial-by-trial decision-making performance, we computed the cross-correlations between behavioral time series, on the one hand, and the dynamics of brain networks, on the other hand. This analysis replicated significant neurobehavioral correlations that peaked at a zero trial lag (Alavash et al., 2017, Figure S2).

We also considered the possible effect of graph thresholding at 10% of network density on dynamic functional connectivity. Toward this aim, we derived the power-envelope coupling strength without thresholding the temporal graphs, and subsequently used raw trial-by-trial measures of functional connectivity in the linear regression analysis. The results were consistent with our main finding: faster perceptual decisions were positively correlated with power-envelope coupling between beta-band neural oscillations (Alavash et al., 2017, Figure S4). This finding suggests that the functional connectivity dynamics of beta-band oscillations are not diminished by fixing the connection density of the temporal brain graphs at 10%. Moreover, our results were also present when brain graphs were thresholded at 5% of network density (Alavash et al., 2017, Figure S8). Finally, to dissociate network from power effects, we implemented a linear regression analysis by adding the trial-by-trial estimates of source power as an additional regressor in the model. This analysis revealed that the network dynamics of beta-band oscillatory power predicted trial-by-trial decision speed over and above the oscillatory source power (Alavash et al., 2017, Figure S5).

In an additional analysis, we investigated the dependence of trial-by-trial network metrics on trial-by-trial acoustic features (i.e., spectral center and stimulus coherence) by means of separate linear regression models (consistent with the main analysis). In each model, we treated the trial-by-trial acoustic features as the dependent variable and tested the significance of the mean regression weights averaged over subjects. On the whole-brain level, we did not observe any consistent significant correlation between brain network metrics and acoustic features in either of the tasks (Alavash et al., 2017, Figure S7). In addition, our main finding—the brain–behavior relation observed on the whole-brain level—was still present when we did not control for the acoustic features of the trial-by-trial stimuli in our regression model. These findings together suggest that the large-scale network organization of coupled beta-band oscillations during auditory perceptual decision-making is not globally altered by the external perturbation induced by stimuli. The global configuration of brain networks, rather, is organized according to the decision goal in light of which the auditory stimuli need to be evaluated.

Overall, our findings show that the dynamics of brain functional connectivity predict trial-by-trial fluctuations in the speed at which auditory perceptual decisions are made and executed. More importantly, faster decisions positively correlated with the ongoing local clustering and modular segregation of large-scale brain networks over trials. At the same time, faster decisions were also predictable from a decrease in the global integration of dynamic brain networks. Brain network correlates of auditory perceptual decision-making were found only for decision speed and were similar across both task sets. Additionally, our findings were specific to the mid-beta band (∼20 Hz) of neural oscillations and were only observed when neural oscillatory responses within both the pre- and poststimulus intervals were used to estimate the trial-by-trial brain network states.

### Regional Network States of Beta-Band Oscillations Predict Decision Speed

The participants judged identical acoustic stimuli under two distinct task sets. Therefore, we not only expected similar network states to correlate with auditory perceptual decision-making in both tasks (mainly associated with frontotemporal cortical communication), but also anticipated finding task-specific network states (potentially emerging from auditory association or higher-order decision areas). On the whole-brain level, we found no significant difference between the two tasks in predicting the trial-by-trial speed or accuracy of auditory perceptual decisions from the ongoing dynamics of brain networks.

However, the regional properties of large-scale brain networks could still predict decision speed specifically in either the pitch or the direction task, or in both, but in different directions. Thus, we aimed to investigate the regional network states that would differentially predict the speed of auditory perceptual decisions during the pitch versus the direction task.

**Figure 5.**
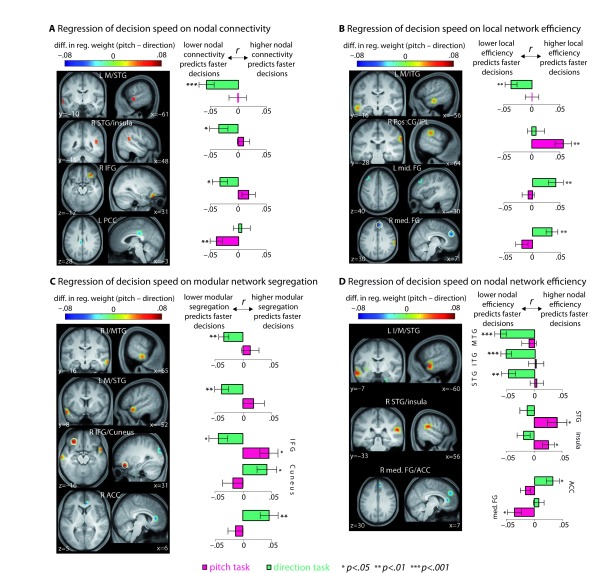
Cortical regions where the network states of beta-band oscillations differentially predicted decision speed during the pitch versus the direction task. At the regional level of large-scale brain networks, we aimed at finding task-specific correlations between the ongoing dynamics of regional network metrics and trial-by-trial decision speed. The analysis was focused on four regional network properties: (A) nodal connectivity, (B) local efficiency, (C) modular segregation (within-module *z*-score), and (D) nodal efficiency. The direction task (green bars), as compared to the pitch task (rose bars), showed stronger correlations with the network properties of sources within temporal and frontal cortices. Within auditory cortex, decreases in local network efficiency (B, first row), modular segregation (C, first two rows), and nodal efficiency (D, first row) supported faster decisions during the direction task. Within the frontal cortex, increases in local efficiency (B, last two rows) and module segregation (C, last row) correlated with faster decisions during the direction task. To create the *color brain maps* for each network metric and source location, the mean regression weights obtained from the direction task data were subtracted from the mean regression weights obtained from the pitch task data. The difference was considered significant if it did not cover the 95% confidence interval of the null distribution generated from shifting the behavioral responses (corrected for the number of source locations using the false coverage statement rate; *p* < 0.05). *Bar plots* show the task-specific mean regression weights (*r*) whose significance was tested against zero by means of one-sample permutation tests with 10,000 repetitions (error bars indicate *SEM*s). L and R abbreviate the left and right brain hemispheres, respectively, and [*x*, *y*, *z*] values indicate MNI coordinates (in millimeters). I/M/STG, inferior/middle/superior temporal gyrus; A/PCC, anterior/posterior cingulate cortex; I/mid./med. FG, inferior/middle/medial frontal gyrus; IPL, inferior parietal lobule.

Figure 5 gives a comprehensive overview of all differential network effects found at the regional level of large-scale brain networks. These maps show significant differential correlations at cortical source locations. Four regional network properties were analyzed (see Alavash et al., 2017): (A) nodal connectivity (also known as *nodal strength*), (B) local efficiency, (C) modular segregation (also known as the *within-module z-score*), and (D) nodal efficiency.

First, the *connectivity* of two network nodes (i.e., MEG source locations) located within the left and right temporal gyri showed significant differential correlations with decision speed during the pitch task in contrast to the direction task (Figure 5A, first and second rows). Lower connectivity at these locations—overlapping with middle and superior divisions of the left and right temporal cortices, respectively—predicted faster auditory perceptual decisions specifically during the direction task. Also, lower connectivity of a network node in right inferior frontal gyrus predicted faster decisions during the direction task (Figure 5A, third row), whereas lower connectivity of a node overlapping with left posterior cingulate cortex predicted faster perceptual decisions during the pitch task (Figure 5A, last row).

Second, lower *local efficiency* of left middle/inferior temporal gyri specifically predicted faster decisions during the direction task (Figure 5B, first row). These faster decisions, however, were concurrent with an increase in local efficiency of the left middle and right medial frontal cortices (Figure 5B, last two rows).

Third, the *modular segregation* of certain network nodes within left and right auditory and frontal cortices showed correlations with decision speed specifically during the direction task (Figure 5C). More precisely, when subjects judged the overall direction of the frequency-modulated tone sweeps faster, two source locations within bilateral auditory cortices showed decreases in their modular segregation (Figure 5C, first two rows). Notably, higher modular segregation of a source location within right anterior cingulate predicted faster decisions during the direction task (Figure 5C; last row).

Finally, we found strong correlations between decision speed during the direction task and the integration of three nodes within left auditory cortex into the whole-brain network (Figure 5D). This result emerged from the lower *nodal efficiency* of three source locations overlapping with left inferior, middle, and superior temporal gyri, and was specific to the direction task. The correlation between decision speed during the direction task and the nodal efficiency of brain networks was not limited to these regions within auditory cortex: higher nodal efficiency of a source location in right anterior cingulate cortex also predicted faster decisions during the direction task (Figure 5D, last row). In contrast, faster perceptual decisions during the pitch task correlated with higher nodal efficiency of right superior temporal gyrus and insula (Figure 5D, second row).

We also investigated the contributions of regional network states to the results observed on the whole-brain level shown in Figure 4. Toward this end, we implemented the same analysis we had done on the whole-brain level, but used a regional network metric extracted per source location over trials as a predictor. This analysis was conducted independently per pitch and direction task. We found significant correlations between the ongoing dynamics of brain regional network metrics and the trial-by-trial speed of auditory perceptual decisions in both the pitch and direction tasks (nondifferential effects; Alavash et al., 2017, Figure S3).

These results were in good agreement with the direction of the correlations observed on the whole-brain level (Figure 4). In brief, higher local network efficiency, modular segregation, and nodal network efficiency of the source locations predominantly within bilateral sensorimotor and parietal cortices predicted faster decisions in both tasks. However, decrease in all of the regional network metrics in source locations predominantly within auditory cortex supported faster decisions, which was more evident in the case of the direction task.

Taken together, the results obtained at the regional level of whole-brain networks point to stronger correlations between brain network states and the speed of perceptual decisions during the direction than during the pitch task. These predictions emerged from auditory and frontal cortices. Among these predictions, the stronger ones (significance levels of *p* < 0.01) converged on decreases in the nodal connectivity, local network efficiency, modular segregation, and nodal network efficiency of regions within auditory cortex. Within the frontal cortex, however, faster decisions during the direction task were predicted by increases in local network efficiency and modular segregation.

## Discussion

A time- and frequency-resolved analysis of large-scale brain networks during auditory perceptual decision-making unveiled two main results: For both the pitch and direction tasks and on the whole-brain level, faster decisions were predicted by higher local efficiency and modular segregation, but lower global integration of coupled beta-band oscillations. On the regional level, the results of our task-differential analysis revealed that the relatively more difficult direction task relied critically on specific network configurations of temporal and frontal regions. We discuss these results in terms of neural oscillations and complex brain networks. Further elaboration is provided in the Supplementary Discussion (Alavash et al., 2017).

### Network Dynamics of Beta-Band Oscillations Predict Decision Speed

Oscillations are key to neural communication (Adrian, 1944; Buzsaki & Draguhn, 2004; Fries, 2015; Schroeder & Lakatos, 2009). Although most studies on neural oscillations aim to uncover mechanisms for dynamic excitation, inhibition, and synchrony (Jensen & Mazaheri, 2010; Keitel & Gross, 2016; Singer & Gray, 1995; Womelsdorf et al., 2007), fewer studies have focused on long-range synchronizations between distributed cortical areas (e.g., Doesburg, Green, McDonald, & Ward, 2009; Donner & Siegel, 2011; Hanslmayr, Staresina, & Bowman, 2016; Varela, Lachaux, Rodriguez, & Martinerie, 2001). Here we measured coupling between the power envelopes of MEG source signals, which has been shown to underlie global network communication across the cortex (Siegel et al., 2012). The temporal network dynamics of these large-scale interactions predicted the speed of auditory perceptual decisions, which was specific to brain networks tuned at the mid-beta band of neural oscillations, centered around 20 Hz.

Beta-band oscillations have classically been associated with sensorimotor functions (Aumann & Prut, 2015; Brovelli et al., 2004; Crone, Miglioretti, Gordon, & Lesser, 1998) and are attenuated during voluntary movements or motor imagery (Pfurtscheller, 2001; Pfurtscheller & Lopes da Silva, 1999; Turella et al., 2016). In our study, faster decisions negatively correlated with the power of neural oscillations within the alpha and beta bands. However, the network effect was specific to the mid-beta band. We argue that this network effect is a manifestation of large-scale neural couplings underlying auditory processing and manual responding. The results of our control analysis suggest that motor actions per se cannot explain all of the correlations we found on the whole-brain level. In this analysis, we used the mid-beta-band data within the period when subjects manually reported their judgments. We observed positive correlations only between decision speed and local network efficiency (Alavash et al., 2017, Figure S1). Knowing that local network efficiency is related to nodal clustering (Rubinov & Sporns, 2010), this effect might be due to local beta-band desynchronization coherently occurring within sensorimotor cortex, thereby forming dense clusters with high local efficiency. Besides our control analysis, we also investigated pre- and poststimulus effects (Alavash et al., 2017, Figure S1), which did not reveal consistent significant effects. These findings together highlight one very important question: what neural dynamics account for predicting decision speed from brain network states?

We here used the correlation between band-limited power envelopes as a functional connectivity measure, and had to choose a certain length for the trial-wise time windows; this is a key parameter in dynamic network analysis (Hutchison et al., 2013) and is related to the frequency content of the underlying signal. Perhaps the length of the above-mentioned time windows (used in control analyses) not long enough to estimate correlations between beta-band power envelopes per trial. Power envelopes evolve within longer time windows than do their underlying carrier frequencies (Siegel et al., 2012). Beta-band power envelopes fluctuate slowly at frequencies below 0.3 Hz (Engel et al., 2013), and therefore their dynamic coupling is estimated better when a time window of ∼3 s (in our case, one trial) is used. Accordingly, our control analysis could not entirely preclude the effect of sensorimotor beta-band desynchronzation in our main findings. Within the last 500 ms of a trial concurrent with planning and executing a manual response, the power envelope of a ∼20-Hz oscillation can be moderately modulated due to the suppression in its underlying carrier. Thus, the trial-wise power-envelope correlations likely reflect the neural couplings underlying perceptual decision-making (from –0.5 to +1 s) and the neural underpinning of manual responses given after the response prompt (from +1 to 1.5 s). Indeed, the effects we found on the regional level support the involvement of auditory, sensorimotor, and frontal cortices in predicting decision speed (Alavash et al., 2017, Figure S2). However, the sluggish dynamics of beta-band power envelopes makes it difficult to dissociate the network states arising from perceptual decision processes from those related to manual responses.

Moreover, previous work on the timing of perceptual decision-making has suggested that, in sensorimotor tasks, a decision is already represented in motor areas before a behavioral response is generated (de Lange, Rahnev, Donner, & Lau, 2013; Gold & Shadlen, 2007; Kelly & O’Connell, 2015). Specifically, a decision variable undergoes a dynamic process through which the accumulated sensory evidence is integrated over time until the action is executed (Schroeder, Wilson, Radman, Scharfman, & Lakatos, 2010; Wyart, de Gardelle,Scholl, & Summerfield, 2012). Several studies across different sensory modalities have associated modulations in beta-band activity with the temporal evolution of perceptual decisions (Senkowski, Molholm, Gomez-Ramirez, & Foxe, 2006). For example, Donner et al. (2007; Donner et al., 2009) reported frontoparietal beta-band activity that was predictive of accuracy during a visual motion detection task, and that only occurred during the decision period of the trials. Accordingly, in Siegel, Engel, and Donner (2011) the authors provided two possible interpretations for these observations: the maintenance and accumulation of sensory evidence during decision formation, or the maintenance of the sensorimotor mapping rule between the accumulated sensory evidence and action (see Engel & Fries, 2010, for an elaboration). Additionally, a study by O’Connell et al. (2012) demonstrated that, during target detection tasks in different sensory modalities, left-hemisphere beta power was modulated by a reduction in the stimulus contrast, and this gradual modulation predicted trial-by-trial reaction times. Finally, the role of beta-band oscillations in decision-making has been supported by animal studies (Haegens et al., 2011; Heekeren, Marrett, & Ungerleider, 2008) and computational modeling (Mostert, Kok, & de Lange, 2015; Sherman et al., 2016).

In sum, and in good agreement with previous accounts (Donner & Siegel, 2011; Hipp et al., 2011), we found that large-scale network interactions mediated by the power of beta-band oscillations are crucial for perceptual decision-making. Our findings draw a direct link between the dynamic network organization of coupled neural oscillations at ∼20 Hz and the trial-by-trial speed of auditory perceptual decisions, built up from early perception to executing manual responses.

### Network States of Frontotemporal Regions Supporting Auditory Perceptual Decisions

Our task-differential analyses at the regional level suggest that the arguably more difficult direction task, in comparison to the pitch task, relied critically on specific network configurations of beta-band oscillations. The differences we found in nodal network topology across the two tasks were specific to MEG sources located within the temporal and frontal cortices. Within the vicinity of auditory cortex, effects in support of faster decisions during the direction task converged toward *decreases* in the (i) nodal connectivity, (ii) local efficiency, (iii) modular segregation, and (iv) nodal efficiency of source locations mostly overlapping with the anterior division of left superior temporal cortex. Within frontal cortex, however, *increases* in (i) the local efficiency of left middle and medial frontal gyri and (ii) the modular segregation of right anterior cingulate predicted faster decisions during the direction task.

One pattern forged of these results is that faster decisions during a particularly challenging auditory perceptual task are accompanied by an increase in the network segregation of frontal regions, and that this segregation supports higher-order decision-related processes. Theoretically, high local clustering of neighbor nodes is associated with high efficiency in local information transfer and fault tolerance (Achard & Bullmore, 2007), indicating how well neighbor nodes can still communicate when the target node (in our case, a frontal region) is removed. As such, when making a perceptual decision is relatively difficult (i.e., the direction task), the decision process benefits from a more autonomous network configuration of frontal regions.

The critical involvement of frontal regions in perceptual decision-making has been supported by previous animal studies on local field potentials (LFPs), in which frontal cortex responses were found to selectively encode auditory stimulus features (Fritz, David, Radtke-Schuller, Yin, & Shamma, 2010) or to show higher synchrony in the beta band (19–40 Hz) as a cortical representation of a task’s rules (Antzoulatos & Miller, 2016; Buschman, Denovellis,Diogo, Bullock, & Miller, 2012). Additionally, in the visual domain it has been shown that top-down control of attention is mediated by higher coherence between frontal and parietal LFPs in the beta band (22–34 Hz; Buschman & Miller, 2007). Recently, Stanley et al. (2016) found that higher local synchrony between LFPs over lateral prefrontal cortex within 16–20 Hz predicted stimulus categorization. Finally, a casual role for frontal cortex in perceptual decision-making was recently proposed by Rahnev, Nee, Riddle, Larson, and D’Esposito (2016).

In contrast to what we observed in frontal regions, network states concurrent with less clustered and less segregated auditory regions were found to speed up decisions during the direction task. One possibility is that during these states brain networks were more integrated (the opposite pole of segregation). However, we did not find significant correlations between decision speed and so-called module participation (Guimerà & Nunes Amaral, 2005), a well-established nodal metric that is quantified on the basis of intermodular connectivity and is attributed to network integration and hubs (Sporns, 2013; Sporns, Honey, & Kötter, 2007; van den Heuvel & Sporns, 2013). Accordingly, the decreases in the clustering and segregation of auditory regions are likely due to pruning of some short-range (intramodular) connections. This local network reconfiguration might be necessary in order to remove direct connections and instead establish longer paths through intermediate critical nodes, thereby supporting the decision process.

We also note that faster decisions during the direction task were predicted by a decrease in the nodal efficiency of left auditory regions. This was perhaps due to the emergence of longer paths between these regions and other network nodes and the pruning of long-range shortcuts. This globally less-efficient and more-distributed information routing might be necessary to support perceptual decisions for the more difficult direction task. Indeed, a study by Siegel, Buschman, and Miller (2015) suggested that, during a sensorimotor decision task, information was not restricted to specific cortical regions, but instead was distributed across graded specialized cortical regions. In addition, and relevant to frequency-specific distributed information routing, a study by Weisz, Wuhle et al. (2014) demonstrated that, during conscious perception, brain networks tuned at 17 Hz get more globally integrated through shorter communication paths.

To conclude, the present study suggests that the large-scale network organization of coupled neural oscillations at ∼20 Hz (the beta band) underlies how quickly momentary auditory perceptual decisions are made and executed. Thus, global communication in brain networks during perceptual decision-making is likely implemented by neural oscillations at around 20 Hz. During auditory perceptual decision-making, this dynamic global communication appears as complex network interactions between beta-band neural oscillations evolving within lower-order auditory and higher-order control areas.

## MATERIALS AND METHODS

### Participants

Twenty healthy, right-handed volunteers (15 females, five males; age range 20–32 years, mean ± *SD* age = 26.2 ± 3.35 years) participated in the study. None of the participants reported any neurological diseases or hearing problems. Ethical approval was obtained from the local ethics committee of the University of Leipzig. All procedures were carried out with written informed consent of the participants and in accordance with the principles of the Declaration of Helsinki. Volunteers received monetary compensation for participating in the study.

### Stimuli

Two auditory task sets were designed that used identical stimuli consisting of variable acoustic textures (see, e.g., Overath et al., 2010, or the “cloud of tones” in Znamenskiy & Zador, 2013) of 400 ms duration (Figure 1A). Each stimulus consisted of 72 frequency-modulated sine tone ramps, or sweeps, of 100 ms duration. The starting time points and frequencies of the individual sweeps were uniformly distributed across time and log frequency. Their frequency slopes spanned ±3.3 octaves per second.

For manipulation of the spectral center (low vs. high), the acoustic textures had one of eight spectral centers relative to a mean center frequency of 707.1 Hz. More specifically, the textures could deviate ±2, 1, 0.5, or 0.25 semitones from the center frequency. Their spectral centers were approximately 630, 667, 687, 697, 717, 728, 749, and 794 Hz.

For manipulation of the spectral coherence, a variable proportion (25%, 50%, 75%, or 100%) of the sweeps were assigned the same frequency slope; that is, they were “coherent” with one another. The rest of the sweeps had randomly assigned slopes.

### Tasks

The participants had to judge one feature of the auditory stimuli under two distinct task sets (Figure 1B). The feature was either the overall spectral center of the sweeps in the acoustic texture (the “pitch” feature: high or low) or the overall direction of the individual sweeps (the “direction” feature: up or down).

Trials started with a white fixation cross that appeared on a black background at the center of a back-projection screen. After a prestimulus interval of 1 s (with no time jitter), an acoustic texture was presented. Then the participants gave a delayed response to the auditory stimulus. This means that 0.6 s after the offset of the acoustic texture, the participants were visually prompted by the decision labels for the left- and right-hand response buttons (see Figure 1B). During the response window, the fixation cross was replaced by a question mark, asking for the subject’s perceptual decision. The response to the previous trial was immediately followed (with no time jitter) by the presentation of the fixation cross for the subsequent trial.

To prevent systematic effects of motor preparation, the mapping of the high/low and up/down labels to the two response buttons was randomized across trials. Participants had 3 s at maximum to respond before the experiment would automatically proceed to the next trial. As in all MEG studies from the Leipzig Max Planck center (e.g., Wöstmann et al., 2016), the auditory stimuli were presented via a nonmagnetic, echo-free stimulus delivery system with almost linear frequency characteristics in the critical range of 200–4000 Hz.

On a separate session before MEG recording, adaptive perceptual tracking was conducted with feedback so that each participant’s average accuracy converged at ∼70% in each task. This was done to avoid ceiling or floor effects, and to assure that the two tasks were challenging enough.

At the recording session, the participants completed eight task blocks, each consisting of 128 trials (∼8 min). The two auditory paradigms, namely the *pitchtask* and the *directiontask*, alternated from block to block, and the initial task was randomized across the participants. Each block contained two trials of each possible combination of direction (two levels), coherence (four levels), and spectral center (eight levels). Subjects were presented two blocks at the beginning to familiarize them with the stimuli and the tasks. The last six blocks were used for data acquisition, resulting in a total of 384 trials per task for each subject. Between the blocks, participants were given self-paced breaks.

### Analysis of Behavioral Data

The performance of the subjects was measured using the average proportions of correct responses (i.e., accuracy) and decision speed (defined as [response time]^−1^). Decision speed was calculated relative to the onset of the response prompt (see Figure 1B) and was used as a proxy for the difficulty of perceptual decision-making during each task. Trials on which no response was given were discarded from the analysis.

To estimate the dynamic pattern of decision accuracy over trials, a moving-average procedure was applied to the trial-by-trial binary responses (i.e., correct = 1, incorrect = 0; rectangular window with unit height and a length of four trials). The choice of the window size was guided by a recent study by Alavash et al. (2016), in which the strongest dynamic coupling of hemodynamic brain networks and behavioral accuracy emerged within time windows of ∼16 s (four trials, in our case). To capture trial-by-trial fluctuations in decision speed, we used inverse response times on trials in which a decision (correct or incorrect) was given. The procedures above gave us time series estimates of decision accuracy and speed over trials per subject (Figure 1C).

To test the correlations between the trial-by-trial estimates of decision accuracy or speed and the trial-by-trial acoustic features (i.e., spectral center and percent coherence) of the auditory stimuli, we used a two-level general linear model (GLM). In this model, the time series estimates of decision accuracy or speed were predicted by an acoustic feature. Since the positively skewed distribution of response times can violate the normality assumption underlying the general linear model (Baayen & Milin, 2010), the dependent variable and the regressors were first rank-transformed and then normalized (i.e., *z*-scored) before estimating the regression models (Cohen & Cavanagh, 2011).

### MEG Data Acquisition and Preprocessing

MEG responses were recorded using a 306-channel Neuromag Vectorview system (Elekta, Helsinki, Finland) in an electromagnetically shielded room (Vacuumschmelze, Hanau, Germany) at a sampling rate of 1,000 Hz with a bandwidth of 330 Hz DC. Movement of each participant’s head relative to the MEG sensors was monitored by means of five head-position measurement coils. An electroencephalogram from 64 scalp electrodes (Ag/AgCl) was recorded but not analyzed in this study.

The raw MEG data were first subjected to the Maxfilter software to suppress any disturbing magnetic interference using the signal space separation method (Taulu, Kajola, & Simola, 2004). Next, the data were corrected for head movements and scanned for intervals during which channels were static or flat. Subsequently, signals recorded from 204 planar gradiometer sensors at 102 locations were fed into the following steps, which were implemented in MATLAB (version 2015a; MathWorks, Natick, MA, USA) using the Fieldtrip toolbox (Oostenveld, Fries, Maris, & Schoffelen, 2011) and other custom scripts.

The signals were high-pass-filtered at 0.5 Hz (finite impulse response [FIR] filter, sync window, order 6,000, zero-phase filtering) and low-pass-filtered at 140 Hz (FIR filter, sync window, order 44, zero-phase filtering). Next, epochs from −1 to +2 s around the onset of the acoustic textures were extracted and down-sampled to 500 Hz.

Independent component analysis was employed to exclude artifactual components containing heart, eye, or muscle activity. Then the single-trial sensor data were visually inspected to exclude trials that still contained eye blinks or movements, muscle activity, static or flat intervals, signal jumps, or drifts, or that had a range larger than 300 pT/m (mean number of rejected trials ± *SEM*: 29.4 ± 4).

### Time-Frequency Analysis of MEG Sensor Data

To analyze the induced perturbation of the MEG signal power during trials, spectrotemporal estimates of the sensor signals were obtained within −0.5 to 1.5 s (relative to the onset of the acoustic textures), at frequencies ranging from 8 to 32 Hz on a logarithmic scale (Morlet’s wavelets; number of cycles =6). The logarithms of the squared magnitudes of the wavelet coefficients were then baseline-corrected relative to the power of the signals within −0.5 to 0 s.

### Source Projection of MEG Sensor Data

Individual forward head models were created on the basis of each participant’s T1-weighted magnetic resonance image (3–T Magnetom Trio, Siemens, Germany). The anatomical images were segmented using Freesurfer and coregistered to the MEG coordinates using the MNE software (http://martinos.org/mne). The fit of approximately 200 digitized head surface points (Polhemus Fastrak 3D digitizer) to the reconstructed head surface was optimized using the iterative closest-point algorithm after the manual identification of anatomical landmarks (nasion, left, and right preauricular points). The individual segmented and coregistered anatomical images were spatially normalized to the standard stereotaxic Montreal Neurological Institute space. The inverses of these operations were applied to a 12-mm grid created in the template brain in order to obtain subject-specific grids in the standard space (1,781 inside-brain source locations separated by 12 mm distance).

To obtain the physical relation between the sources and sensors for all grid points, single-shell volume conduction models (Nolte, 2003) were constructed using the individual segmented anatomical images. The weakest of three dipole orientations per grid point was removed. Next, a linearly constrained minimum-variance beam-forming approach (Van Veen, Van Drongelen, Yuchtman, & Suzuki, 1997) was implemented. Spatial adaptive filters were generated by first concatenating all single-trial signals into one time series per subject, and then computing the covariance matrices using these time series. The regularization parameter was set to 7% and the singular-value decomposition approach was used to estimate the dominant dipole orientation independently per grid point. Finally, the single-trial sensor data from −1 to +2 s around the onset of the acoustic textures were multiplied by the spatial filters, and the results were treated as trial-wise source-projected signals in the subsequent analyses.

### Time-Frequency Analysis of Source-Projected Signals

Time–frequency representations of the source-projected signals were derived using Morlet’s wavelets based on multiplication in the frequency domain. As in Hipp et al. (2012), the spectral band-width of the wavelets was set at 0.5 octaves (number of cycles = 6). The center frequencies were spaced logarithmically using base 2, with exponents ranging from 3 to 5 in steps of 0.25. In addition, we included three lower frequencies at 1, 2, and 4 Hz in order to thoroughly investigate the neurobehavioral correlations within the range 1–32 Hz. Note that since our analysis was done for each trial (see below), to capture the low-frequency oscillations per trial the time–frequency estimations at 1, 2, and 4 Hz were accomplished by mirror-symmetric extension of the source signals to the left and right.

For the main analysis, time points from −0.5 to 1.5 s (relative to the onset of the acoustic textures), in steps of 0.05 s, were used to extract complex-valued spectrotemporal estimates of the source-projected signals per trial (41 data points). For analysis of the prestimulus interval, time points from −0.85 to 0 s (relative to the onset of the acoustic textures) were used. For analysis of the poststimulus interval, we analyzed the time points from the onset of the acoustic textures up to +1 s, during which the participants listened to the auditory stimuli but did not manually give their responses. Additionally, since the power of beta-band oscillations is known to be related to the preparation and execution of movements (Aumann & Prut, 2015; Brovelli et al., 2004; Crone et al., 1998), we performed one control analysis. That is, we analyzed the data within the response window (+1 to +1.5 s relative to the onset of the acoustic textures) at frequencies within the beta band (16–28 Hz). This analysis was aimed at investigating possible effects arising from the neural processes involved in the button press rather than in auditory perceptual decision-making. The results of these analyses are summarized in supplementary Figure S1.

### Correlation Between Ongoing Neural Oscillatory Power and Auditory Perceptual Decision-Making

On a trial-by-trial basis, in order to predict individuals’ decision-making performance from the ongoing power of the source-projected signals over trials, we implemented a two-level GLM approach. First, at the single-subject level, time series estimates of trial-by-trial decision accuracy or speed (Figure 1C) were predicted by the baseline-corrected power of the whole-brain source-projected signals over trials, while controlling for effects of the acoustic features (i.e., spectral center and percent coherence). For this analysis, the time points from −0.5 to 1.5 s (relative to the onset of the acoustic textures), in steps of 0.05 s, were used to extract complex-valued spectrotemporal estimates of the source-projected signals per trial. Subsequently, for each pitch and direction task, separate GLMs were constructed. This procedure was applied to the data obtained from each participant at each frequency of neural-oscillatory power (1–32 Hz). To account for the normality assumption underlying the GLM (Baayen & Milin, 2010), the dependent variable and the predictors were first rank-transformed and then normalized (i.e., *z*-scored) before estimating the regression models (Cohen & Cavanagh, 2011). Finally, the regression weights obtained from the fit of each GLM per task were averaged over participants at each frequency and statistically compared with a null distribution of mean regression weights.

### Power Envelope Correlations and Functional Connectivity Analysis

The power envelope of a band-limited oscillatory signal is the squared magnitude of the time–frequency signal following wavelet decomposition. To assess frequency-specific neural interactions, we computed Pearson’s correlations between the log-transformed powers of all pairs of sources per trial (Figure 2). This analysis was done at each frequency within the range 1–32 Hz.

To eliminate the trivial common covariation in power measured from the same sources, we used the orthogonalization approach proposed by Hipp et al. (2012) prior to computing the power correlations (see Alavash et al., 2017). This approach has been suggested and used to circumvent overestimation of instantaneous short-distance correlations, which can otherwise occur due to magnetic field propagation (Mehrkanoon, Breakspear, Britz, & Boonstra, 2014; Siems, Pape, Hipp, & Siegel, 2016).

The procedure above gave us frequency-specific *N*-by-*N* functional connectivity matrices (where *N* denotes the number of source locations) per subject and trial, for each pitch and direction task (Figure 2).

### Building Dynamic Brain Networks

To construct brain graphs from the functional connectivity matrices, different approaches have been suggested and used (Fornito, Zalesky, & Breakspear, 2013; Garrison, Scheinost, Finn, Shen, & Constable, 2015; van Wijk, Stam, & Daffertshofer, 2010). One way is to construct brain graphs over different network densities by including links in the graph according to the ranks of their absolute correlation values (Alexander-Bloch et al., 2010; Ginestet, Nichols, Bullmore, & Simmons, 2011). In our study, to make the brain graphs comparable in terms of size across subjects and trials, the number of links in each brain graph per trial was fixed at 10% of the network density. The choice of the density threshold was based on previous work demonstrating that the brain network correlates of behavior are observed within a low-density range of network connections (Achard & Bullmore, 2007; Alavash, Hilgetag, Thiel, & Giessing, 2015; Alavash et al., 2016; Giessing, Thiel, Alexander-Bloch, Patel, & Bullmore, 2013; Godwin, Barry, & Marois, 2015). However, to assure that the results were not specific to one network density, we repeated our analysis at 5% of the network density, and have summarized these results in supplementary Figure S8.

Subsequently, binary undirected brain graphs were built, from which graph-theoretical network metrics were extracted per trial (Figure 2). The mean functional connectivity was estimated as the mean of the upper-diagonal correlation values within the sparse temporal connectivity matrices.

### Network Diagnostics

Three key topological properties were estimated per trial: *mean local efficiency*, *network modularity*, and *global network efficiency*. These graph-theoretical metrics were used to capture dynamic patterns of functional integration and segregation on the local, intermediate, and global scales of network topology, respectively (Figure 2). For each graph-theoretical metric, we computed a global network diagnostic that collapsed the metric into a single measure on the whole-brain level, and a regional diagnostic characterizing the same metric but for a certain cortical source location. The regional network diagnostics were therefore used to localize the cortical regions contributing to the network integration or segregation measured on the whole-brain level. First, we used nodal connectivity (also known as nodal strength) as the regional measure of mean functional connectivity. Second, local efficiency was computed within each source’s neighborhood graph as the regional measure of mean local efficiency. Third, module segregation (which was quantified using the “within-module *z*score”) was used to measure the strength of the membership of a given source in its network module. Finally, nodal efficiency was measured to capture the integration of a given source into the entire network (see supplementary information, Alavash et al., 2017, for details).

### Correlation Between Brain Network Dynamics and Auditory Perceptual Decision-Making

To predict trial-by-trial perceptual decision-making performance from the ongoing brain network states, we employed the same regression approach we had used for predicting performance from the brain’s ongoing oscillatory power. That is, we implemented a GLM in which time series estimates of trial-by-trial decision accuracy or speed (Figure 1C) were predicted by the graph-theoretical network metrics over trials (Figure 2), while controlling for effects of the acoustic features (i.e., the spectral center and percent coherence). For each network diagnostic, a separate GLM was constructed. Thus, each model consisted of three regressors together with a constant term. Since the statistical distribution of the temporal brain network metrics is not necessarily normal, the dependent variable and the predictors were first rank-transformed and then normalized (i.e., *z*-scored) before estimating the regression model (Cohen & Cavanagh, 2011). This procedure was applied separately to the data obtained from each pitch and direction task, subject, and frequency of neural oscillatory power. Finally, the normalized regression weights obtained from the fit of each GLM per task were averaged over participants at each frequency and statistically compared with a null distribution of mean regression weights.

To measure the task specificity of the correlations between a given network diagnostic and either trial-by-trial decision accuracy or speed, we also computed the mean difference between the regression weights *β* obtained from each GLM model—that is, mean(*β*_pitch_ − *β*_dir_).

### Statistical Analysis

#### Behavioral data

Mean decision speeds and average accuracies were compared between the different task and stimulus conditions by means of an analysis of variance (ANOVA) for repeated measures, using Task (pitch, direction), Coherence (four levels), and Spectral Center (four levels) as within-subjects factors. We used generalized eta-squared ($\eta_{G}^{2}$; Bakeman, 2005) as the effect size statistic. Prior to the ANOVA, the distributions of the behavioral measures across participants were statistically analyzed by means of bootstrap Kolmogorov–Smirnov tests with 10,000 repetitions to ensure that the data derived from a normally distributed population. The bootstrap Kolmogorov–Smirnov test, unlike the traditional Kolmogorov–Smirnov test, allows for the presence of ties in the data (Sekhon, 2007). The behavioral measures (i.e., mean decision speed and average accuracies) were compared between the pitch and direction tasks using exact permutation tests for paired samples. The correlations between each of the behavioral measures across the two tasks were tested using the rank-based nonparametric Spearman’s *ρ* correlation (Spearman, 1904) with 10,000 permutations applied to the correlation coefficients (Pesarin & Salmaso, 2010). To test the correlation between the trial-by-trial estimates of decision accuracy or speed and the trial-by-trial acoustic features of the stimuli, a two-level GLM was applied separately to each pitch and direction task per subject. The regression weights obtained from the fit of each GLM were averaged over participants and statistically tested against zero using one-sample exact permutation tests.

#### Neurobehavioral correlations

To predict trial-by-trial perceptual decision accuracy or speed from the ongoing brain oscillatory power or network states, we implemented a two-level GLM approach. The regression weights obtained from the fit of each GLM were averaged over participants at each frequency and statistically compared with a null distribution. The null distribution was generated using a randomization procedure in which the trial-by-trial binary responses or decision speeds were circularly shifted 350 times (the number of trials remained after preprocessing in every subject) over trials, per task and per subject. Circular shifting preserves the autocorrelation structure inherent to the time series (e.g., the trial-by-trial sequential correlation in response times; Baayen & Milin, 2010), and thus has the advantage over random shuffling. For the circularly shifted behavioral responses, we conducted the same analysis steps we had done for the empirical behavioral data. To statistically test the significance of the neurobehavioral correlations, the observed mean regression weight was compared with the null distribution generated from the randomization procedure at each frequency. The observed mean regression weight was considered significant if it was higher than the 97.5th percentile or lower than 2.5th percentile of the null distribution (upper and lower bounds of the horizontal shading in Figures 3 and 4).

#### Regional analysis

At the regional level of the brain networks, we tested whether the time series estimates of decision-making performance under each pitch and direction task differentially correlated with the regional diagnostics of the brain networks (see Alavash et al., 2017). Toward this end, the mean regression weights obtained from the direction task data were subtracted from the mean regression weights obtained from the pitch task data. The significance of the difference between the correlations was statistically tested using a null distribution of the difference in mean regression weights generated from circularly shifting the behavioral responses. We also investigated the regional network states per pitch and direction task separately. Toward this end, we implemented the same analysis we had done on the whole-brain level, but used a regional network property estimated per source location over trials. The results of this analysis are summarized in supplementary Figure S2.

#### Significance thresholds

For all statistical tests (i.e., inference on the behavioral and brain-network effects) we used *p* < 0.05 (two-sided) as the threshold of significance. For the analysis on the whole-brain level, in order to correct for the multiple comparisons entailed by the number of frequency bins (14), we implemented a correction method suggested by Benjamini and Yekutieli (2005) and used by Obleser, Leaver, Vanmeter, and Rauschecker (2010) and Obleser and Weisz (2012). In this method, called the “false coverage statement rate” (FCR), we first selected those frequencies at which the observed mean regression weight did not cover the null distribution at the confidence level of 95%. In a second correction pass, we (re)constructed the FCR-corrected confidence intervals for these selected frequencies at a level of $1-F_{s}\times \frac{q}{F_{t}}$, where *F*_*s*_ is the number of selected frequencies at the first pass, *F*_*t*_ is the total number of frequency bins tested, and *q* is the tolerated rate for false coverage statements, here 0.05. The FCR correction procedure yields inflated, and thus more conservative confidence limits (bounds of the horizontal shading in Figure 3). To correct for the multiple comparisons entailed by the regional analysis, we used the same procedure to adjust the confidence limits according to the number of source locations (1,781).

## ACKNOWLEDGMENTS

This research was supported by the Max Planck Society (through a Max Planck Research Group grant to J.O.) and the European Research Council (ERC Consolidator AUDADAPT, Grant No. 646696 to J.O.). Yvonne Wolff and Burkhard Maess helped acquire the MEG data.

## AUTHOR CONTRIBUTIONS

Mohsen Alavash: Conceptualization; Formal analysis; Investigation; Methodology; Visualization; Writing – original draft; Writing – review & editing Christoph Daube: Data curation; Formal analysis; Visualization; Writing – review & editing Malte Wöstmann: Conceptualization; Methodology; Writing – review & editing Alex Brandmeyer: Conceptualization; Data curation; Methodology; Visualization; Writing – review & editing Jonas Obleser: Conceptualization; Funding acquisition; Methodology; Project administration; Resources; Supervision; Writing – review & editing

## TECHNICAL TERMS

Neural oscillation: A rhythmic voltage fluctuation generated by the electrical activity of neuronal populations and characterized by its dominant frequency
Large-scale network: A network comprising widely distributed nodes showing short- and long-distance associations
Perceptual decision-making: Evaluation—often implicit—of sensory information in order to arrive at a behaviorally relevant decision
Topology: The wiring diagram of a set of interacting elements, representing how their associations are organized as a whole
Network modularity: Segregation of a network into groups of partner nodes showing dense intraconnections but sparse interconnections
Network integration: The capacity of a network to combine distributed information across its nodes, often characterized by global network efficiency
Functional connectivity: Statistical dependency between time-varying brain activities
Graph-theoretical network analysis: Mathematical formalization aimed at studying systems of interacting elements (nodes) with pair-wise associations (connections)
Acoustic texture: An auditory object composed of a dense overlay of multiple, frequency-modulated tone sweeps
Network efficiency: A graph-theoretical measure inversely related to the average length of the shortest paths existing between network nodes
